# Oxidative Etching of Hexagonal Boron Nitride Toward Nanosheets with Defined Edges and Holes

**DOI:** 10.1038/srep14510

**Published:** 2015-09-29

**Authors:** Yunlong Liao, Kaixiong Tu, Xiaogang Han, Liangbing Hu, John W. Connell, Zhongfang Chen, Yi Lin

**Affiliations:** 1 National Institute of Aerospace, 100 Exploration Way, Hampton, VA, 23666, USA; 2 Department of Chemistry, Institute for Functional Nanomaterials, University of Puerto Rico, Rio Piedras Campus, San Juan, Puerto Rico, 00931, USA; 3 Department of Materials Science and Engineering, The University of Maryland, College Park, MD, 20742, USA; 4 Advanced Materials and Processing Branch, NASA Langley Research Center, Hampton, VA, 23681-2199, USA; 5 Department of Applied Science, The College of William and Mary, Williamsburg, VA, 23185, USA

## Abstract

Lateral surface etching of two-dimensional (2D) nanosheets results in holey 2D nanosheets that have abundant edge atoms. Recent reports on holey graphene showed that holey 2D nanosheets can outperform their intact counterparts in many potential applications such as energy storage, catalysis, sensing, transistors, and molecular transport/separation. From both fundamental and application perspectives, it is desirable to obtain holey 2D nanosheets with defined hole morphology and hole edge structures. This remains a great challenge for graphene and is little explored for other 2D nanomaterials. Here, a facile, controllable, and scalable method is reported to carve geometrically defined pit/hole shapes and edges on hexagonal boron nitride (h-BN) basal plane surfaces via oxidative etching in air using silver nanoparticles as catalysts. The etched h-BN was further purified and exfoliated into nanosheets that inherited the hole/edge structural motifs and, under certain conditions, possess altered optical bandgap properties likely induced by the enriched zigzag edge atoms. This method opens up an exciting approach to further explore the physical and chemical properties of hole- and edge-enriched boron nitride and other 2D nanosheets, paving the way toward applications that can take advantage of their unique structures and performance characteristics.

Although ideal two-dimensional (2D) nanosheets are infinite, those in the real world are of limited size bounded by peripheral edge atoms. Compared to the basal plane surface atoms, the number of atoms at the nanosheet peripheral edges is often much fewer especially for a larger sheet. However, these atoms are still critical to many properties of the nanosheet as a whole, such as electronic structure, chemical reactivity, and catalytic activity. Therefore, controllable synthesis of desirable edge structure is of key importance in tailoring the properties and performance of a 2D material, but remains a great challenge^1^,^2^.

A popular type of 2D nanosheets with rich edge atoms is nanoribbons that have narrow widths but high lateral aspect ratio. Nanoribbons are known to have significantly different electronic properties from their nanosheet counterparts due to quantum confinement in one lateral dimension as well as the presence of edge atoms^3^,^4^. Another emerging type of nanosheet with large amount of edge atoms is vacancy-populated ones. One such example that has received significant attention recently is “holey graphene”^5^,^6^,^7^, sometimes also called “graphene nanomesh” or, in computation models, “graphene antidot”^8^. For these 2D materials, in addition to peripheral sheet edges, they also possess edges around the high density vacancies/holes which can have a pronounced impact on the properties of nanosheets, making these materials perform differently from their intact counterparts. For example, the presence of holes in holey graphene opens up a gap at the Dirac point, making them useful for field effect transistors with large on-off ratios^9^,^10^,^11^. The presence of abundant hole edge atoms also made these materials advantageous in sensing applications^12^,^13^. The morphology of the holes, which is defined by the hole edges, may also give rise to diverse properties that can be exploited for applications. For example, it was recently demonstrated that the holes on graphene provide less tortuous through-plane transport pathways for electrolyte ions, resulting in high charge/discharge rates^14^ as well as high volumetric performance in energy storage^15^,^16^. Although it has yet to be systematically demonstrated, it can be expected that modification of the hole edge chemical structures can be used in the fine tuning of through-plane molecular transport. Moreover, the epitaxial growth of in-plane seamless graphene-boron nitride heterojunctions has been achieved starting from basal plane-etched graphene^17^,^18^,^19^. Such in-plane hybrid nanosheets are expected to have unique electronic properties, further highlighting the importance of controlling the shape, concentration and chemistry of edge structures in 2D nanomaterials.

In comparison to graphene which consists of a single type of atom, heteroatomic 2D nanomaterials, such as hexagonal boron nitride (h-BN) and molybdenum disulfide (MoS_2_), have more diversity in terms of the edge structure and chemistry. For example, edges in h-BN (Supplementary Information Figure S1) can be in the form of armchair (*A*), which is terminated by B-N bonds, or zigzag (*Z*). A zigzag h-BN edge can be either all B-terminated (*Z*_B_) or all N-terminated (*Z*_N_). The values of chirality angles can be used to determine the neighboring edge structures. For example, an angle of 120° indicates neighboring *A*-*A* or *Z*_B_-*Z*_N_ edges, while an angle of 150° indicates neighboring *A*-*Z*_B_ or *A*-*Z*_N_ edges (Figure S1).

Similar to the case of holey graphene, the presence of arrays of through-plane holes with enriched edge atoms on such derivatives of 2D heteroatomic nanosheets could also enable unique applications such as catalysis, through-plane transport, and molecular sensing. While various chemical methods were demonstrated to obtain holey graphene in large quantities^5^,^6^,^7^,^8^,^9^,^10^,^11^,^12^,^13^,^14^,^15^,^16^, scalable approaches to synthesize etched heteroatomic nanosheets such as boron nitride (BNNSs) have been less explored. It is well known that controlled electron beam irradiation on BNNSs creates well defined vacancy defects with predominately triangle-shaped *Z*_N_ edges^20^,^21^. It was proposed that the higher kinetic displacement rate of B compared to that of N atoms under electron bombardment might be the dominating mechanism to form *Z*_N_ edges^22^. In another report, Cun *et al.* showed the penetration of low energy Ar ions underneath Rh-supported single-layer h-BN “nanomesh” (*i.e.*, nanosheets with periodic corrugated surfaces due to Rh-BN interactions), which subsequently induced the formation of 2-nm holes upon annealing^23^. Very recently, Li *et al.* demonstrated the strong oxidation resistance of mechanically exfoliated monolayer and few-layered BNNS in air. The oxidized BNNS exhibited elongated and randomly oriented etched pits and paths on the nanosheet surfaces^24^.

Most of the above experiments were conducted on a substrate scale. For many applications, large quantities of materials are usually needed which require more scalable synthetic approaches. Thus far, achieving both hole/edge structural fidelity and scalability for 2D nanosheets remains elusive even for graphene that has been extensively studied. In this article, a simple and effective oxidative etching process is reported to selectively obtain pits and holes of various defined geometrical shapes with ordered edge structures on pristine h-BN crystalline platelets. The oxidized platelets can be subsequently purified and exfoliated to obtain BNNSs with similar structural motifs. The method is potentially scalable to obtain large amounts of hole/edge-defined nanosheets which may enable various applications.

## Results

In the experiments, metallic Ag nanoparticles were used as the etching catalyst and were grown on both basal plane surfaces and peripheral edges (hereafter referred to as “perimeters” to avoid confusion) of h-BN platelets using a facile and scalable solvent-free process previously developed by the authors (see **Methods** and Figure S2)^25^. The as-obtained Ag-decorated h-BN (Ag-BN; Ag:BN ~ 1:5 mol/mol) sample was subjected to heating in static air using an open-ended tube furnace at an elevated temperature. The selected temperature range for the etching was identified using thermogravimetric analysis (TGA; Figure S3). The TGA trace showed that there was very little weight gain (i.e., an indication of the oxidation of h-BN into heavier B_2_O_3_ with N_2_ evolution) for the pristine h-BN when dynamically heated to ~1000 °C in air. However, with the presence of Ag nanoparticles, a significant weight gain was observed at ~820 °C, suggesting a much lower oxidation threshold. This is likely due to a catalytic effect, similar to that observed in the carbon gasification in catalytic preparation of holey graphene from Ag-decorated graphene that was reported previously by the authors^26^. Temperatures of 800, 900, and 1000 °C were then selected as the oxidation temperatures in the following studies described herein. The samples were all heated to the target temperature at 10 °C/min and held isothermally for 3 h unless otherwise specified.

**Figure 1:**
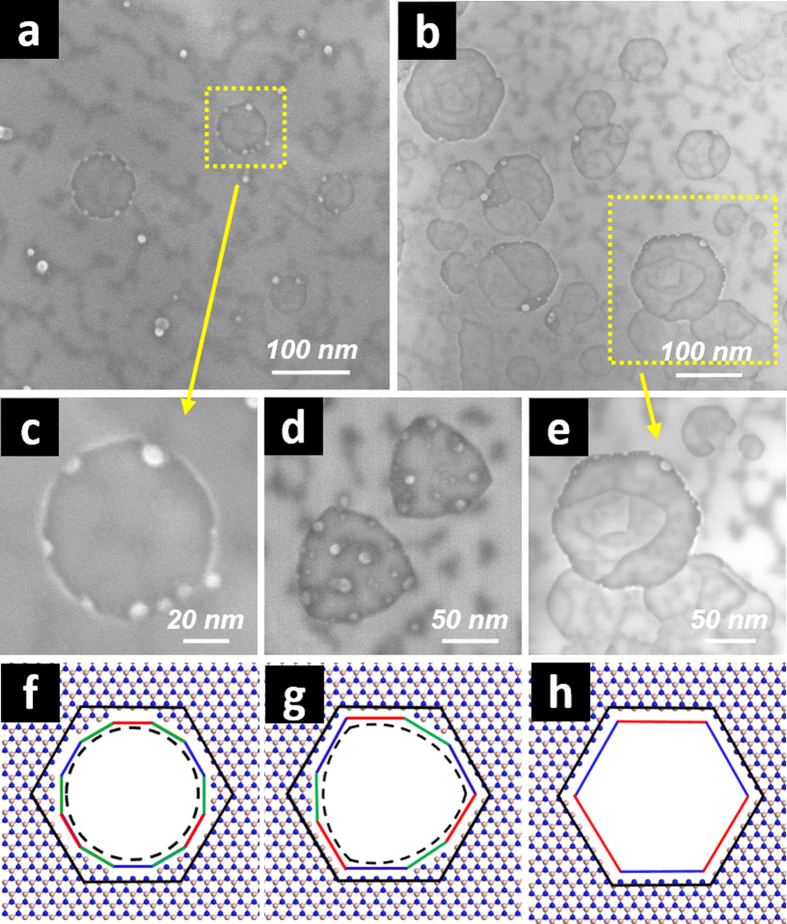
Pit formation on h-BN basal plane due to Ag-catalyzed etching at 800 °C for 3 h. (**a**) and (**b**) are SEM images of the h-BN basal plane surface at lower magnifications where multiple circular and hexagonal pits formed, respectively. (**c**–**e**) are close-up SEM images of pits with shapes of a circle, Reuleaux triangles, and a hexagon, respectively. (**f**–**h**) are the corresponding atomic models for (**c**–**e**). There are other possible structures for (**d**,**e**), which are shown in Figure S4 in Supplementary Information. *Z*_B_–, *Z*_N_– and *A–* edges are marked with red, blue, and green lines, respectively.

As shown in the scanning electron microscopy (SEM) images in Fig. 1, the basal plane of h-BN platelets in the Ag-BN sample heated to 800 °C exhibited shallow pits, with lateral dimensions of ~50–100 nm and depths of a few atomic layers (more discussions below). A variety of pit shapes were observed, including circles (Fig. 1a,c), Reuleaux triangles (Fig. 1d), and hexagons (Fig. 1b,e). Circular pits indicated that the etching was mostly isotropic with little preference of a certain type of edge chirality. Closer examination indicated their polygonal nature (Fig. 1c). A good atomic model for circular/polygonal pits is a dodecagonal hole with a 3-fold symmetry (Fig. 1f; 12 sides total; internal angle: 150°). The repeating edge ensemble was of *Z*_B_-*A*-*Z*_N_-*A* type structure. Interestingly, this exact structure was observed upon further etching at a higher temperature, which will be discussed in a later section. Pit shapes of Reuleaux triangles are visual approximations of nonagons, which are also of a 3-fold symmetry (9 sides total; internal angles: 120°, 150°). The structure of the 3 repeating edge units could be either *Z*_B_-*A*-*Z*_N_ (“Reuleaux_z_”; Fig. 1g) or *A*-*Z*_*B/N*_-*A* (“Reuleaux_a-B_” or “Reuleaux_a-N_”; Supplementary Information Figure S4a & b). Hexagonal pits (6 sides total; internal angle: 120°) should have either 3 repeating units of *Z*_B_-*Z*_N_ (Fig. 1h; “hexagon_z_”; 3-fold symmetry) or all *A* edges (Figure S4c; “hexagon_a_”; 6-fold symmetry). These various types of pit/hole features have an interesting structural correlation. For example, the dodecagonal and the nonagonal holes could be viewed as “truncated” hexagonal holes at all 6 and 3 corners, respectively, with extra basal plane atoms at those corners. The hexagons before truncation are indicated in Fig. 1f–h. It is thus plausible that one of the oxidative etching pathways could have occurred in the sequence of circular (i.e., entirely isotropic)—dodecagon—nonagon—hexagon.

Note that nonagons were only rarely seen, perhaps indicating their meta-stable nature in comparison to the other observed hole shapes. In addition, it is interesting that no equilateral triangle shapes were observed in all catalytic etching conditions investigated in this report. As mentioned in the introductory remarks, those shapes are signatures of B- or N- vacancy defects (3-fold symmetry; *Z*_N_ and *Z*_B_-edged, respectively; Figure S4d & e) on the BNNS basal plane as a result of electron beam irradiation^20^–^22^. Their absence suggests that their formation is not thermodynamically favorable under the catalytic oxidation conditions. This might also relate to the unfavorable occurrence of the nonagonal pits, which are geometrically close to the equilateral triangles. Another noticeable observation was that the same sheets typically exhibited pit shapes of similar geometry with the same orientation (e.g., Fig. 1a,b), consistent with long range h-BN basal plane crystallinity. This also suggests that there is a fairly homogeneous driving force toward the formation of one certain type of pit shape across each platelet surface.

The majority of these newly formed pits had Ag nanoparticles attached to the edges, consistent with a catalytic oxidation mechanism. In a control experiment, a neat h-BN sample without Ag decoration remained stable at 800 °C and no etching was observed. Note that the catalytically active Ag nanoparticles were of much smaller sizes (<10 nm) than those in the starting Ag-BN sample (Figure S2), which is possibly due to high temperature-induced migration, disintegration, and redistribution of Ag nanoparticles. In fact, the majority of the Ag nanoparticles seemed to have aggregated and became detached from the h-BN surface. Nevertheless, the appearance of the Ag nanoparticle-attached pits indicates that the depth of the pits should be very close to and no greater than the size of these attached nanoparticles. Therefore, the estimation of the pit depth is ~5–10 nm, equivalent to ~15–30 atomic layers.

The pits presumably originated from the pre-existing surface defects of h-BN where a small amount of Ag nanoparticles preferentially remained anchored despite the heat-induced disintegration and migration of the majority of the Ag particles. The average pit density was not high (roughly <3 pits per 100 × 100 nm^2^), consistent with the highly crystalline nature of the h-BN starting material.

**Figure 2:**
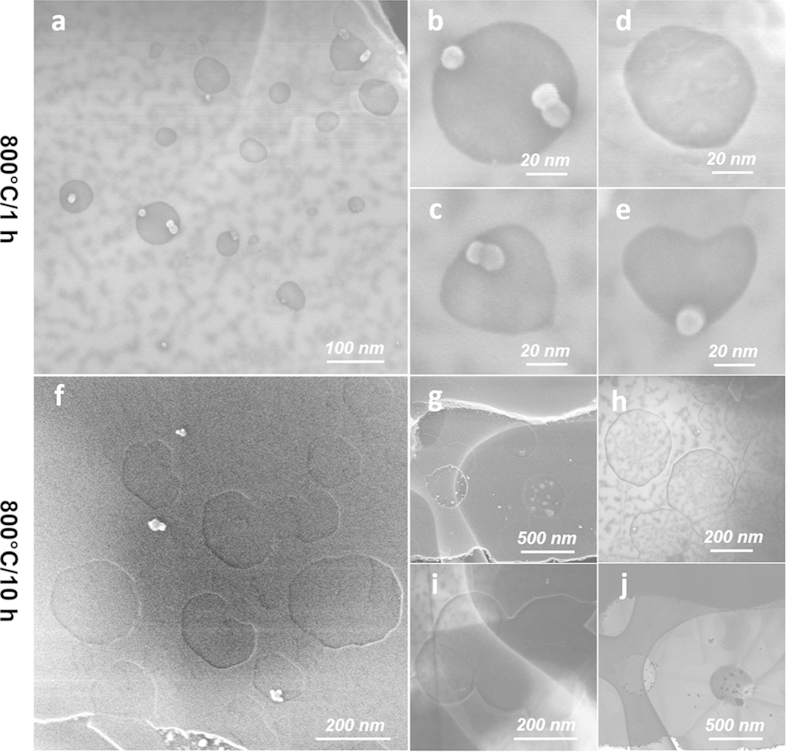
Catalytic pit growth on h-BN at 800 °C with different heating durations. (**a**–**e**) 1 h; (**f**–**j**) 10 h. (**a**–**i**) are SEM images at various magnifications; (**j**) is a TEM image showing an exfoliated sheet with multiple wrinkles and pits.

The formation and growth of the pits was a progressive process. With shorter heating duration of 1 h at 800 °C, the shapes of the pits were not only smaller (~20–80 nm), but also more isotropic (i.e., more circular than polygonal) than those found with 3 h heating (Fig. 2a–e). With 1 h heating duration, some very irregular shapes were also occasionally found. Longer heating duration at the same temperature resulted in larger pits. For example, by heating at 800 °C for 10 h, polygonal pits with dimensions of 200–400 nm were prevalent (Fig. 2f–h). In some cases, neighboring pits merged to form larger (Fig. 2h) and more irregular shaped structures (Fig. 2i). A statistical chart summarizing the evolution of the pit shapes formed at 800 °C but with various heating time is given in Figure S5a.

Moreover, many platelets from 10 h heating in the microscopy specimens became qualitatively thinner since they appeared more transparent under electron beam in transmission electron microscopy (TEM), while those from pristine h-BN were entirely dark (i.e., impenetrable to electrons). The h-BN sheet shown in Fig. 2j even exhibited wrinkles and folds, which are morphological features only found in well-exfoliated nanosheets that became laterally flexible due to reduced thickness^27^. These microscopy specimens were prepared by dispersing the solid samples in methanol with minimal bath sonication (<15 s) to maximally retain the sample morphology. Apparently after long duration heating at 800 °C, the etching significantly weakened the interlayer interactions so that such short term, ultra-mild sonication was sufficient to exfoliate some of the etched h-BN platelets.

**Figure 3:**
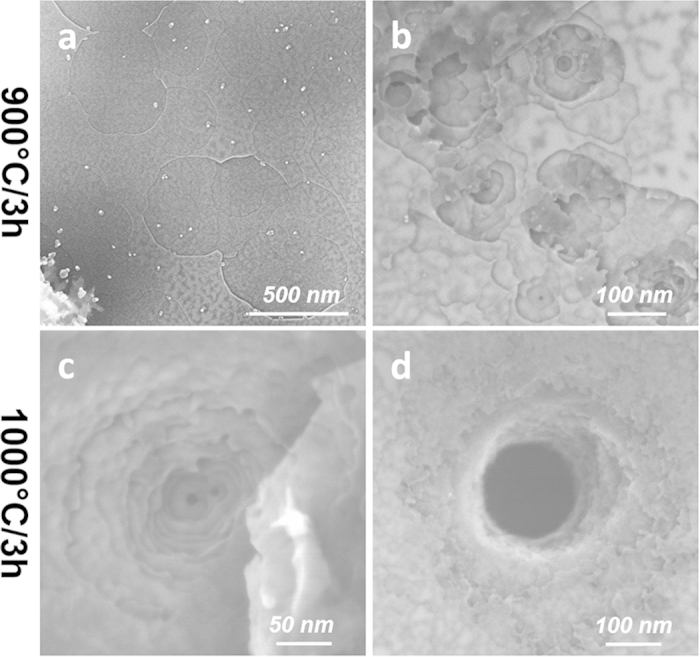
Catalytic pit growth on h-BN at (a,b) 900 °C and (c,d) 1000 °C, each for a duration of 3 h. (**a**) shows enlarged shallow pits; (**b**,**c**) show pits penetrating into deeper layers; (**d**) shows a through-thickness hole.

Similar pit-enlarging and sheet thinning effects were also observed for samples oxidized at higher temperatures (e.g. 900 °C for 3 h, Fig. 3a). In addition, with increasing reaction temperature, the etching at some locations took place into deeper layers with gradually decreasing pit diameters (Fig. 3b,c). At 1000 °C, even through-thickness holes were observed (Fig. 3d). Noticeably, the edges of these hierarchically layered pits or holes were very rough with no defined shapes. However, by soaking these samples with dilute nitric acid overnight at room temperature (see **Methods** for details), the rough morphology was removed, revealing rather smooth and much better defined pit or hole shapes.

**Figure 4:**
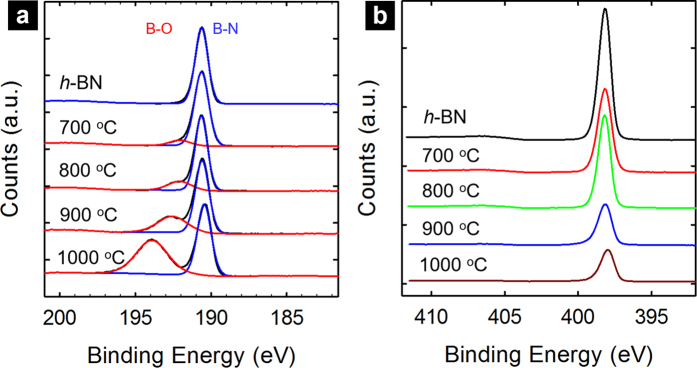
(**a**) B 1 s and (**b**) N 1 s regions of XPS spectra of various Ag-BN samples after 3 h oxidative etching in air at 700, 800, 900, and 1000 °C. The spectra of the pristine h-BN sample were also shown for comparison.

Before entering into more details of the pit/hole shapes and edge structures, a few comments are provided to address the structure of as-etched Ag-BN and the effects of the acid soaking process. First of all, since h-BN is stable in acid, the facile removal of the rough and irregular species at the pit/hole edges suggested that they were most likely increasing buildup of oxidized boron residue that did not evaporate during the reaction. This was supported by both X-ray photoelectron spectroscopy (XPS) and fourier-transform infrared spectroscopy (FT-IR) analyses. For XPS, there was a gradual increase of oxygen content from the survey data (Supplementary Information Table S1) as well as the oxidized boron species (192–193.9 eV in binding energy) (Fig. 4a)^28^. Comparably, the N 1 s spectra (Fig. 4b) exhibited no meaningful change for samples after etching except for decreased intensities, consistent with the expectation that the oxidation of h-BN would result in oxidized boron species (solid but evaporable) and evolution of nitrogen gas so that the remaining N atoms in the solid essentially retained their original chemical bonding with neighboring B atoms.

**Figure 5:**
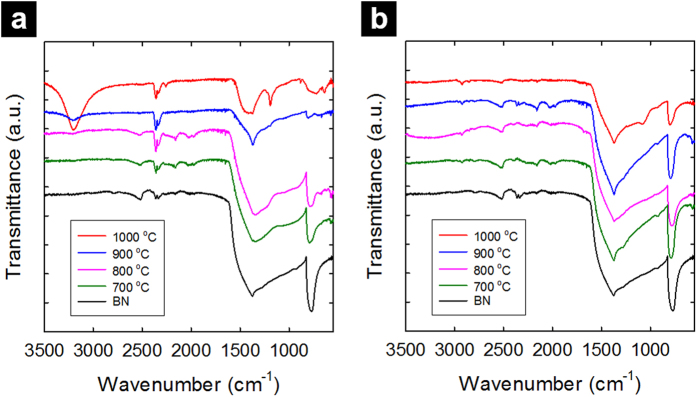
FT-IR Spectra of various Ag-BN samples after 3 h oxidative etching in air at 700, 800, 900, and 1000 °C in comparison with that of pristine h-BN: (**a**) as-etched samples; (**b**) after nitric acid purification.

In FT-IR, the as-etched Ag-BN samples (Fig. 5a) also exhibited signals related to oxidized boron species [B-O stretching at 900–1100 cm^−1^, 1230 cm^−1^, (B-)O-H stretching at 3200 cm^−1^] that became more significant with higher temperature oxidation. These signals largely disappeared after acid soaking (Fig. 5b). The structure of h-BN was recovered, as shown by typical B-N stretching and bending modes at 1375 and 770 cm^−1^, respectively, comparable to the starting h-BN sample.

Dilute nitric acid is known to readily dissolve Ag nanoparticles even under ambient conditions^25^. The removal of Ag from the oxidized Ag-BN samples was confirmed by their absence in the microscopy images of those after acid soaking. Therefore, the nitric acid treatment was a facile one-step purification process for the oxidized Ag-BN products.

Note that FT-IR spectrum of the Ag-BN sample from 800 °C etching exhibited rather weak signals from oxidized boron, most of which were likely produced by the preferential oxidation of the nanosheet perimeter (more discussions later). This suggested that there was very little oxidized boron buildup at the pits and their geometrical shapes were very close to their intrinsic structure (Figs 1 and 2). This was shown by the SEM images from a sample after acid soaking (Supplementary Information Figure S6), where the purified h-BN platelets were free of Ag nanoparticles and exhibited pit shapes and sizes that are similar to those of the as-produced sample.

**Figure 6:**
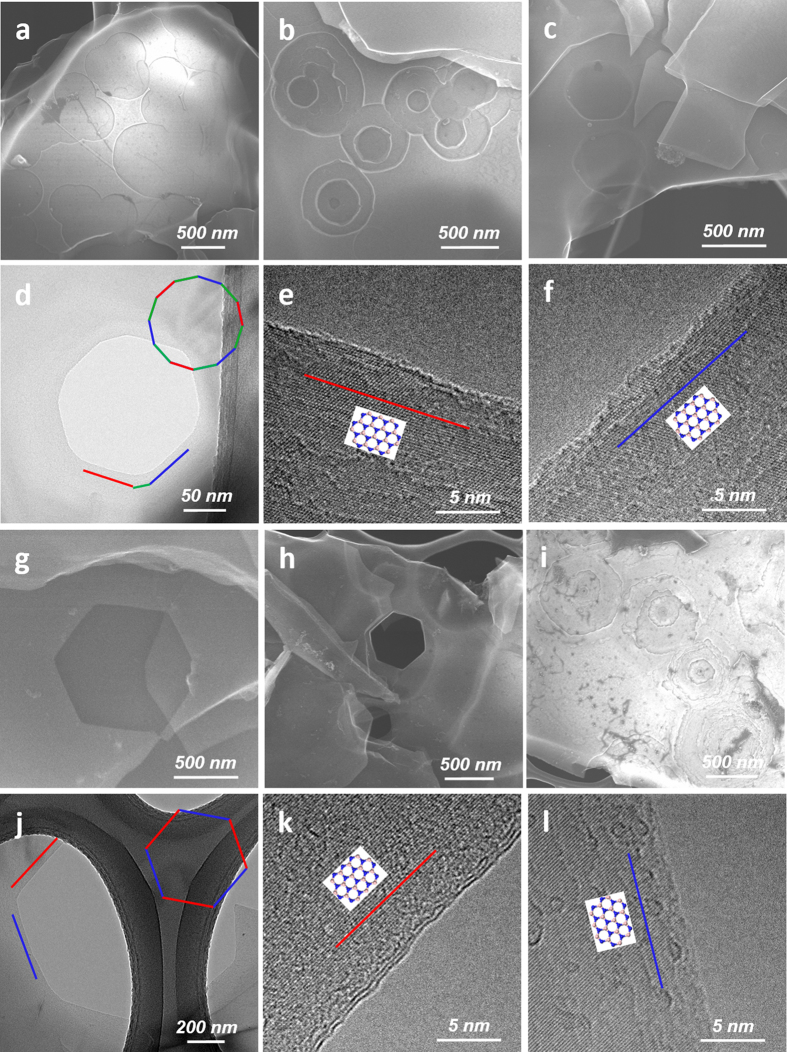
Acid-purified Ag-BN samples from etching at (a–f) 900 °C and (g–l) 1000 °C, respectively. (**a**–**c**) and (**g**–**i**) are SEM images from as-purified samples, while (**d**–**f**) and (**j**–**l**) are TEM images for DMF-exfoliated BNNSs after purification. Red and blue colors of the lines represent edges with *Z* orientation (red for *Z*_B_ and blue for *Z*_N_ or vice versa, because the microscopy resolution was insufficient to differentiate B vs. N), while the green color represents edges with *A* orientation. (**e**,**f**) are high resolution views of the respective areas in (**d**), while (**k**,**l**) are the same for (**j**). Again, the atomic structures shown were only to indicate these edges were all *Z* orientations, but the assignment of B- or N- terminations were interchangeable (either red for *Z*_B_ and blue for *Z*_N_, or vice versa).

The intrinsic (*i.e.*, by removal of excessive oxidized boron) shapes of the pits and holes for the Ag-BN samples oxidized at 900 and 1000 °C were revealed after acid purification. As shown in Fig. 6a–c, polygon-shaped pits, either large and shallow or smaller but penetrating into deeper layers, were present in the samples etched at 900 °C. After 1000 °C treatment, the observed shapes for both pits and through-thickness holes became much more enriched with hexagons (Fig. 6g–i). A statistical chart summarizing the evolution of pit/hole shapes by heating to 800, 900, and 1000 °C (all for 3 h) is shown in Figure S5b.

It was of significant interest to investigate the atomic structure of the edges of these defined shapes. However, most acid-purified etched h-BN platelets were too thick to allow sufficient electron beam transmission for structural investigation. Therefore, the acid-purified etched h-BN samples were subjected to a well established solvent exfoliation process by sonicating the powdery samples in *N*,*N*′-dimethylformamide (DMF) for 6 h to obtain a stable dispersion of exfoliated etched BNNSs (see **Methods** for experimental details)^29^. The BNNSs were of much reduced thicknesses but largely retained lateral dimension. The nanosheets also contained both pits and through-thickness holes, whose shapes were inherited from those in the as-purified sample before exfoliation. Some nanosheets exhibited a rather high density of holes resembling the structure of holey graphene^5^,^6^,^7^,^8^,^9^,^10^,^11^,^12^,^13^,^14^,^15^,^16^, which may be called “holey BNNS” (Supplementary Information Figure S7). However, as a result of higher crystallinity, the density of the holes of the etched BNNS in this work was usually much lower than typical holey graphene sheets.

With sufficiently thin BNNSs, high-resolution TEM (HR-TEM) studies were carried out to investigate the edge structures of the holes. A dodecagon-shaped hole from the exfoliated BNNS sample etched at 900 °C is shown in Fig. 6d. Evaluation of the typical hexagonal lattice packing of h-BN at higher resolution (each bright dot represents a hexagonal ring in the h-BN lattice) revealed that the longer edges were of zigzag direction (Fig. 6e,f). As indicated in the atomic model earlier (Fig. 1f), in an ideal situation, the nearest two *Z* edges should be all terminated by B and N atoms, respectively (i.e., *Z*_B_ and *Z*_N_ edges), with an *A*-edge in between. It should be noted that the fine structures of these edge terminations were likely defective and deviated from such ideal illustrations. Therefore, the chirality assignments of the edge terminations in this study should be more accurately described as the assignments of geometric orientations of the hole edges, which were unambiguous at the current image resolution.

**Figure 7:**
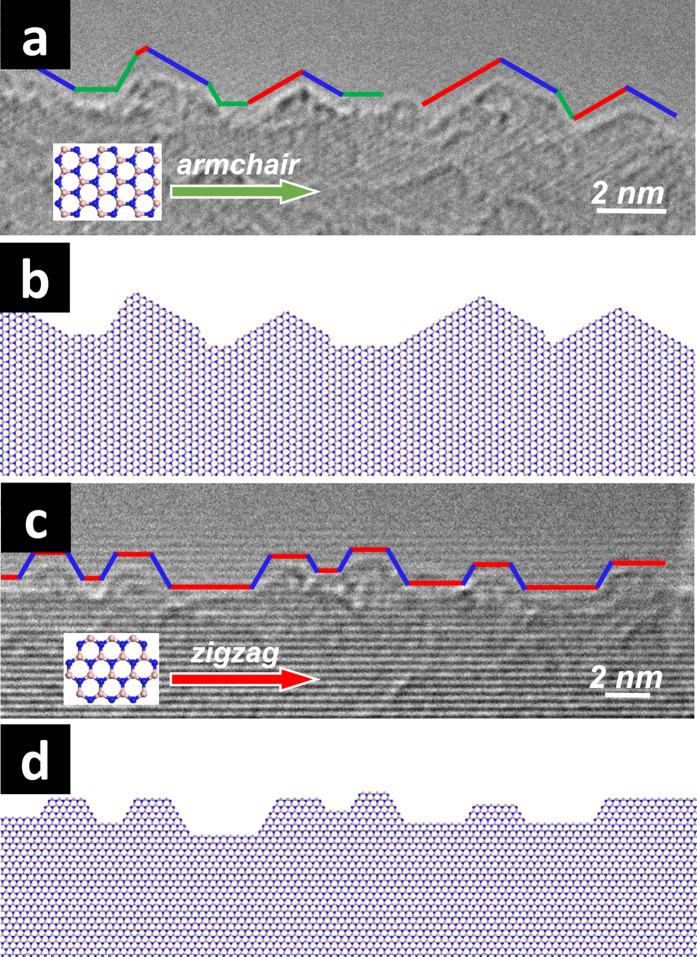
Fine atomic edge structures of hexagonal holes of exfoliated BNNS from purified Ag-BN sample etched at 1000 °C/3 h: (a,c) HR-TEM images and (b,d) th**e** corresponding atomic structural schematics. (**a**,**b**) an edge with *A* orientation exhibiting mostly triangularly-tipped fine structures, which are mostly *Z* terminations. *Z*_B_-, *Z*_N_- and *A*-terminated edges are marked with red, blue, and green lines, respectively; (**c**,**d**) an edge with *Z* orientation exhibiting hexagonally-tipped fine structures, which are all *Z*-terminated. Note the instrument resolution was insufficient to differentiate *Z*_B_ vs. *Z*_N_, thus the assignments of *Z*_B_- and *Z*_N_-terminated edges are interchangeable with *Z*_N_ and *Z*_B_-terminated edges, respectively.

For exfoliated BNNS from 1000 °C etching, as shown in Fig. 6j–l, a regular hexagonal hole (with 3 atomic layers shown in Fig. 6k) exhibited all *Z*-oriented edges (thus with neighboring *Z*_B_-*Z*_N_ pairs), which almost perfectly match the illustration in Fig. 1h. While *Z*-oriented edges were found to be in dominance (8 out of 11 hexagons examined), hexagonal holes with all edge lines of *A* orientations were also found (atomic model illustrated in Figure S4c). Interestingly, closer examination of the finer atomic structure at these *A*-oriented edges revealed many sharp triangular tips with the sides forming 30° or 150° angles with the main *A* orientation, indicating they were *Z*-terminated edges (Fig. 7a). In comparison, the edge lines of *Z* orientations discussed above exhibited hexagonal tips (Fig. 7b), suggesting the local atomic edges were also *Z*-terminated. These findings strongly suggested that the *Z*-oriented and *Z*-terminated edges were both preferred over *A* ones under the oxidative etching conditions in this work. In addition, although the resolution of the HR-TEM equipment used in this work was insufficient to reveal the atomic identities of the edge atoms, it was nevertheless clear that both *Z*_B_ and *Z*_N_ edges were present and stably co-exist as neighboring edges of the pits and holes (at 120° angle, if one edge is *Z*_B_, the neighboring edges must be *Z*_N_). The observation of enrichment of *Z*-oriented and *Z*-terminated edges seems to coincide with a variety of previous experimental studies^30^,^31^,^32^,^33^ and energy calculations^34^ on 2D BN nanomaterials. For example, recent progress in chemical vapor deposition (CVD) preparation of BNNSs^30^ showed that the nanosheets in the initial stage of growth tend to take the shape of triangles with defined *Z* edges. Evidence from unzipping of BN nanotubes (BNNTs) also suggested preference of *Z* orientations along the ribbon lengths^32^,^33^.

**Figure 8:**
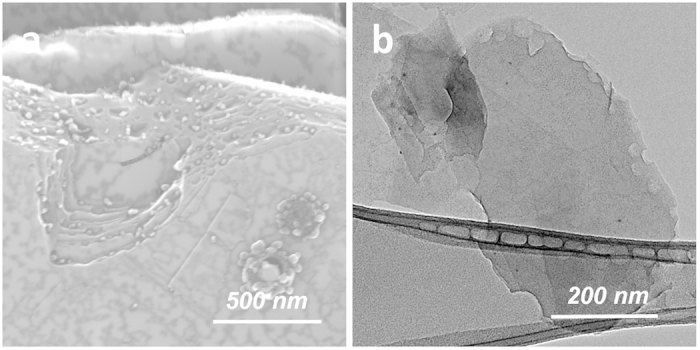
Selective perimeter etching at 700 °C/3 h: (a) as-oxidized Ag-BN sample showing exclusive perimeter etching with a step-like morphology; (b) purified and exfoliated etched BNNSs exhibiting a unique perimeter-etched morphology.

It is of general interest to investigate the difference in etching reactivity of basal plane vs. perimeter edge of h-BN. For this purpose, the oxidation of Ag-BN was conducted at a lower temperature of 700 °C for 3 h. Under this condition, pristine h-BN platelets with originally smooth edges (thus no chirality preference) were almost exclusively etched at the perimeter while the basal plane remained intact (Fig. 8a; Supplementary Information Figure S8a–c). Most etched features contained individual Ag nanoparticles, again suggesting the active catalytic role of Ag during etching. Individual Ag nanoparticleswere predominately seen on the platelet perimeter, while most of those on the basal plane surface formed large agglomerates, in contrast to their well separated forms in the starting Ag-BN sample (Figure S2). The different forms of Ag nanoparticles were likely because the nanoparticles on the basal plane surfaces were of lower anchoring strength than those at the perimeter, and were more prone to heating-induced migration, disintegration, and redistribution. The dimensions of the etched features at the perimeter of h-BN platelets were typically in the range of ~20–50 nm. Most of these features exhibited a partial circular or polygonal shape, suggesting dominating isotropic or near-isotropic etching. An additional observation was that the etching effect seemed to be more pronounced for outer layers, which resulted in a step-like perimeter morphology (Figure S7a–c) that was absent in the pristine h-BN platelets. The oxidized Ag-BN product could be similarly purified using nitric acid and exfoliated using DMF to form a stable dispersion of BNNSs preferentially etched at edges (Fig. 8b). HR-TEM results (Figure S8d–f) indicated both isotropic and polygonal etched perimeter features.

Similar to the oxidative boron buildup observed in basal plane etching, the perimeter of the h-BN platelets became more severely etched and exhibited rough and irregular morphology at 800 °C and above (Supplementary Information Figure S9). After acid purification, partial circles and polygons with smoother edges and dimensions of ~100–300 nm (much larger than those found at 700 °C) were revealed for the sample from 800 °C oxidation (Figure S9d and g). In comparison, the platelet perimeter from oxidation at 900 (Figure S8e) and 1000 °C (Figure S9f) followed by acid purification appeared almost featureless, which could be readily attributed to the overlap from continuous size increase of the above-mentioned individual etched features. DMF-exfoliated BNNS from these samples exhibited similar motifs of newly formed edge features at the perimeter.

**Figure 9:**
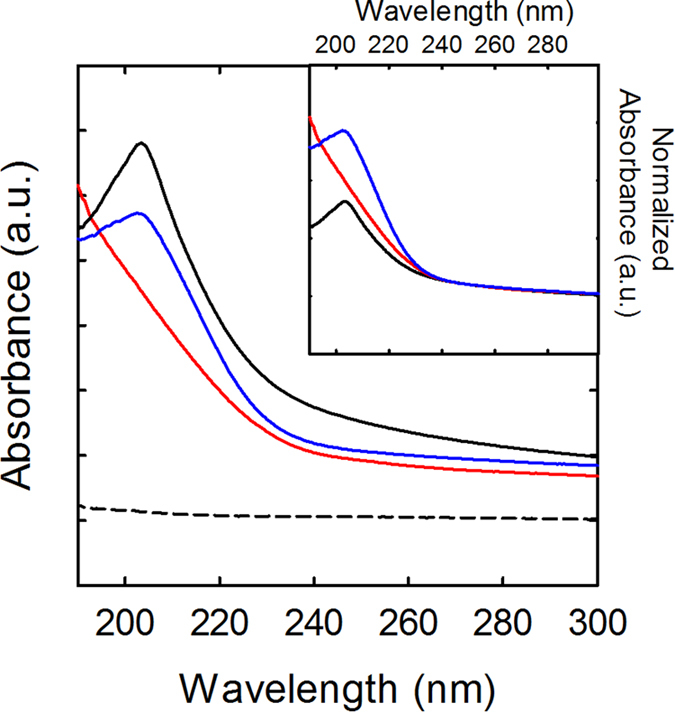
UV optical absorption spectra of as-obtained BNNS aqueous solutions (concentrations on the order of ~0.1 mg/mL) from Ag-BN etched at 900 (blue) and 1000 °C (red) followed by acid purification and exfoliation using water. Water-exfoliated pristine BNNSs (black, solid) and an aqueous solution of B_2_O_3_ with a concentration of 10 mg/mL (black, dashed) are shown for comparison. Inset shows the same three BNNS spectra after normalization.

Finally, the optical properties of edge-enriched BNNSs from exfoliation of the etched h-BN materials should be addressed. The optical absorption spectra of exfoliated BNNSs from etching at 700–900 °C (water was used for the exfoliation to aid in the observation of bandgap in the UV region^35^) revealed the presence of a shoulder on the higher energy side of the typical bandgap peak of water-exfoliated pristine BNNSs (203.5 nm, or 6.09 eV) (Fig. 9). For the BNNSs obtained from etching at 1000 °C, the absorptivity at incident light wavelengths of <240 nm (or >~5.2 eV) was still qualitatively comparable to that of water-exfoliated pristine BNNSs. Surprisingly, the bandgap peak was completely absent (or moved to higher energy beyond the instrument limit 190 nm or 6.53 eV). This was first thought to be due to oxygen doping or residual oxidized boron species; however, the absorption of pure B_2_O_3_ was found to be very weak (also shown in Fig. 9), indicating oxygen doping was not the cause for the high UV absorption and absence of a bandgap peak. Nevertheless, the above results serve as strong evidence that the electronic properties of the etched BNNSs were significantly modulated, most likely a result of increasingly enriched *Z* edges. In a previous report, it was also shown that BN nanoribbons also have different optical properties from parent BNNTs due to the presence of enriched edge atoms^36^. Raman spectra results suggested less pronounced sensitivity to above electronic modulation, with the spectra of the same exfoliated etched BNNSs appearing quite similar to that of the exfoliated pristine BNNSs (Supplementary Information Figure S10). There was only a small upshift (~0.7 cm^−1^) of the *E*_2g_ peak for the sample from 1000 °C etching, which could be a result of oxygen doping^24^.

## Conclusions

A facile Ag-catalyzed etching method to carve defined shapes and edges on h-BN basal plane surface is reported. The etching was a progressive process that initially occurred isotropically, then in chirality-defined orientations that were dependent on the temperature and time duration^37^. It was also observed that lower reaction temperature enabled selective etching at the platelet perimeter with the basal plane remaining intact. Upon purification and exfoliation, the resultant etched BNNSs with much reduced thicknesses inherited the hole and edge structural motifs and exhibited significant change in electronic properties as indicated from optical absorption spectra. This method opens up an exciting scalable approach to further explore the properties and applications of BNNSs with defined holes and edges for potential applications such as catalysis and molecular transport/separation. It can be envisioned that this synthetic strategy can be further extended to the synthesis of other edge/vacancy-enriched 2D nanomaterials as a means to further explore their structure/property relationships and identify applications that can take advantage of their unique structures and performance characteristics.

## Methods

### Materials

h-BN powder (size -10P, Lot HZ010PA4.$06) was provided by UK Abrasives. Silver acetate (99%) and DMF were purchased from Aldrich. All chemicals and solvents were used as received.

### Characterizations

SEM and low-magnification TEM imaging were carried out using a Hitachi S-5200 field-emission SEM system at an accelerating voltage of 30 kV. HR-TEM experiments were conducted on a JEOL 2100 field emission TEM system at an accelerating voltage of 200 kV. Raman spectroscopy was performed using a Thermo-Nicolet-Almega dispersive Raman spectrometer with 532 nm excitation. Optical absorption spectra were obtained using a Perkin-Elmer Lambda 900 UV/vis/NIR spectrometer. FT-IR spectra were acquired on a Thermo-Nicolet FT-IR 300 spectrometer equipped with a Thunderdome Swap-Top single reflection attenuated total reflectance (ATR) module. XPS data were obtained on a Kratos Axis 165 X-ray photoelectron spectrometer operating in hybrid mode using monochromatic Al KR X-rays (1486.7 eV). TGA data was collected on a Netzsch TG 209 F1 Libra thermogravimetric analyzer at 10 °C/min in air.

### Synthesis of Ag-BN

In a typical experiment, h-BN powder (1 g, 42 mmol) were dry mixed with powdered silver acetate (1.4 g, 8.4 mmol) using a mortar and pestle until homogeneous (about 5–10 min) under ambient conditions. The solid silver acetate/h-BN mixture was then transferred to an aluminum pan and heated in a nitrogen oven (Blue M Electric A-5245-Q Inert Gas Oven) to 350 °C over 1 h and held isothermally for 3 h. The nitrogen flow was typically at 60–100 mL/min. Upon cooling to room temperature, the product was collected as the final Ag-BN sample with Ag:BN ratio of 1:5 mol/mol.

### Oxidative Etching of Ag-BN

In a typical experiment, a Ag-BN sample (100–200 mg) was placed in a ceramic or quartz crucible and heated in air by using an open-ended tube furnace (MTI Model GSL-1100X-UL) to a given temperature (in the range of 700–1000 °C in this work) at a ramp rate of 10 °C/min and held isothermally for a certain period of time (1–10 h in this work). Upon cooling to room temperature, the product was collected as the final etched Ag-BN sample.

### Purification of Etched h-BN

In a typical experiment, excess dilute HNO_3_ (14%, 20 mL) was added to an as-etched Ag-BN powder sample (100 mg) in a round-bottom flask and stirred for 24 h under room temperature. The slurry was then centrifuged at ~3000 × g for 10 min. The top solution was decanted to obtain the residue, which was washed with deionized water via several repeated centrifugation-decantation processes. The sample was dried in a vacuum oven overnight to obtain purified, etched h-BN as a white-colored powder.

### Exfoliated, Etched BNNS

In a typical procedure, a purified, etched h-BN sample (~20 mg) was sonicated in DMF (20 mL) for 6 h using a bath sonicator (Bransonic Model M2800H or Model B2510-MTH, both 40 kHz). The reaction flask was capped with a rubber stopper to avoid loss of volatile reagents. Upon completion of sonication, the mixture was subjected to centrifugation at 3000 × g for 10 min to separate the supernatant dispersion from the residue. The supernatant was collected as the exfoliated, etched BNNS. Similar experiments were also carried out using water as the exfoliation solvent for optical absorption studies.

## Additional Information

**How to cite this article**: Liao, Y. *et al.* Oxidative Etching of Hexagonal Boron Nitride Toward Nanosheets with Defined Edges and Holes. *Sci. Rep.* **5**, 14510; doi: 10.1038/srep14510 (2015).

## Supplementary Material

Supplementary Information
