# Supplementary material for: Oxidative Etching of Hexagonal Boron Nitride Toward Nanosheets with Defined Edges and Holes
Source: Sci Rep. 2015 Sep 29;5:14510. doi: 10.1038/srep14510 (PMC4586441; doi:10.1038/srep14510)
Supplement: Supplementary Information [file srep14510-s1.pdf]

--Supplementary Information--

# Oxidative Etching of Hexagonal Boron Nitride Toward Nanosheets with Defined Edges and Holes

**Yunlong Liao,[1,2] Kaixiong Tu,[2] Xiaogang Han,[3] Liangbing Hu,[3] John W. Connell,[4] Zhongfang Chen,[2,*] Yi Lin[1,5,*]**

[1] National Institute of Aerospace, 100 Exploration Way, Hampton, VA, 23666, USA
[2] Department of Chemistry, Institute for Functional Nanomaterials, University of Puerto Rico, Rio Piedras Campus, San Juan, Puerto Rico, 00931, USA
[3] Department of Materials Science and Engineering, University of Maryland, College Park, MD, 20742, USA
[4] Advanced Materials and Processing Branch, NASA Langley Research Center, Hampton, VA, 23681-2199, USA
[5] Department of Applied Science, The College of William and Mary, Williamsburg, VA, 23185, USA
Corresponding Authors: yi.lin@nianet.org; zhongfangchen@gmail.com

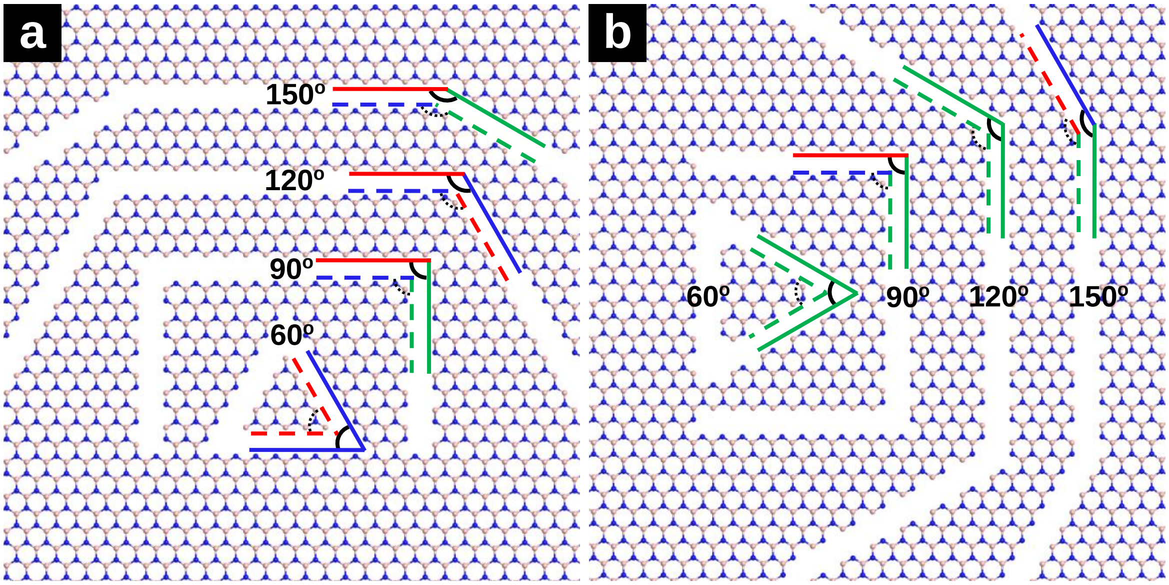


**Figure S1.** Schematic diagrams showing atomic structures of neighboring edges of BNNS with various chirality angles. Internal (corresponding to vacancy defects, pits, or holes) and external edges (corresponding to perimeter edge) are marked with solid and dashed lines, respectively. $Z_B$, $Z_N$ and $A$ edges are marked with red, blue, and green lines, respectively. For chirality angles of 90° and 150°, $Z$ (= $Z_B$ + $Z_N$) and $A$ edges are of equal probability. For chirality angles of 60° and 120°, however, the neighboring edges are either both $Z$ (a) or both $A$ (b). At 60°, the neighboring Z edges are either both $Z_B$ or both $Z_N$. At 120°, the neighboring Z edges are of alternative termination ($Z_B$-$Z_N$ or $Z_N$-$Z_B$).

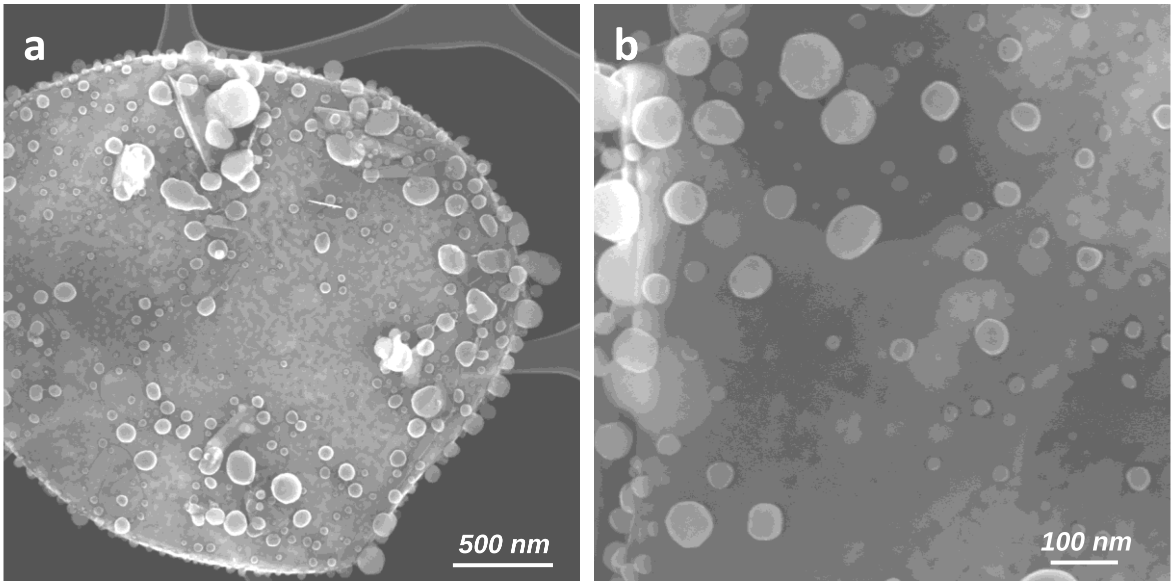


**Figure S2.** Typical SEM images of Ag-BN at (a) low and (b) high magnifications, respectively.

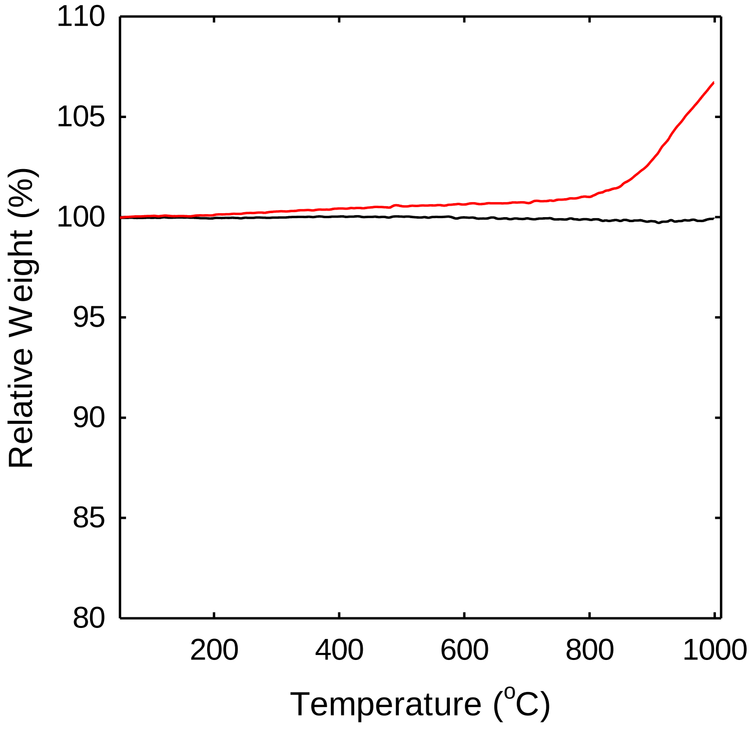


**Figure S3.** TGA traces of Ag-BN (red) and pristine h-BN (black) samples at a heating rate of 10 °C/min in air.

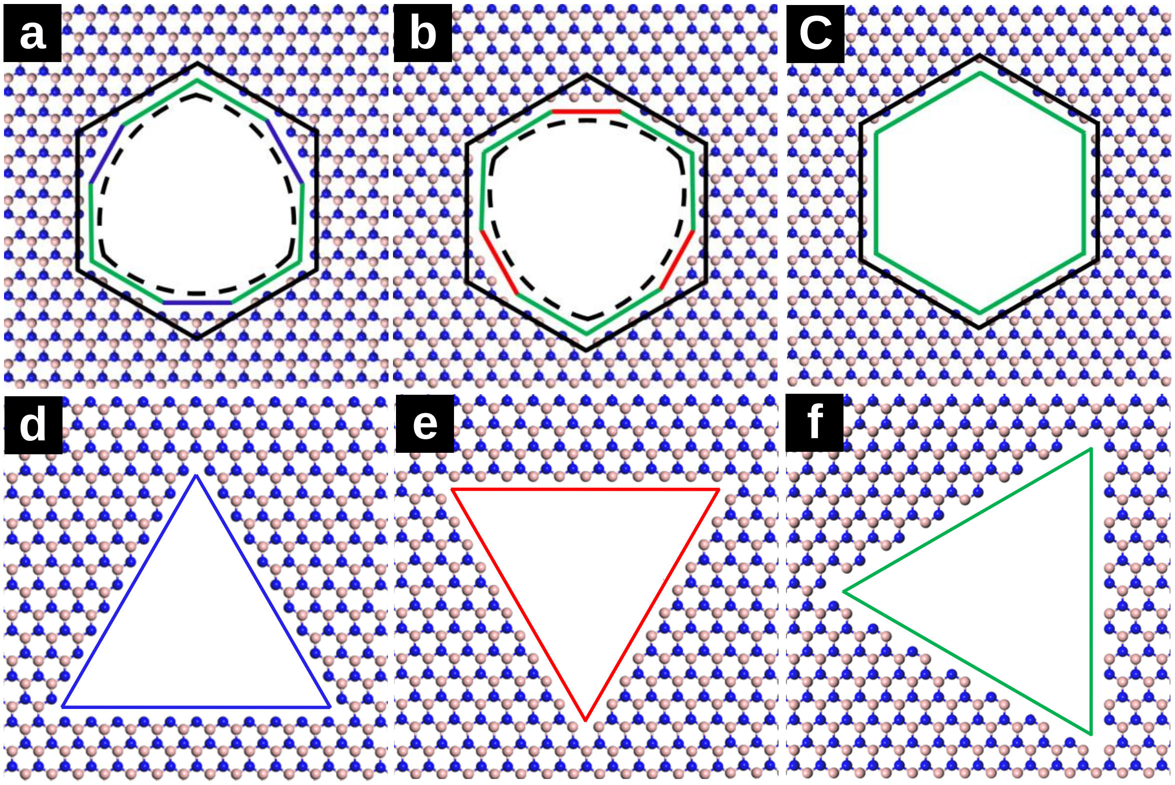


**Figure S4.** Atomic models of possible hole edge structures on BNNS. (**a**) a nonagon (visually "a Reuleaux triangle" as indicated by dashed curves) with $A$-$Z_N$-$A$ repeating edge ensembles; (**b**) a nonagon with $A$-$Z_B$-$A$ repeating edge ensembles; (**c**) a hexagon with all $A$ edges; (**d**) a triangle with all $Z_N$ edges; (**e**) a triangle with all $Z_B$ edges; and (**f**) a triangle with all $A$ edges. $Z_B$, $Z_N$ and $A$ edges are marked with red, blue, and green lines, respectively.

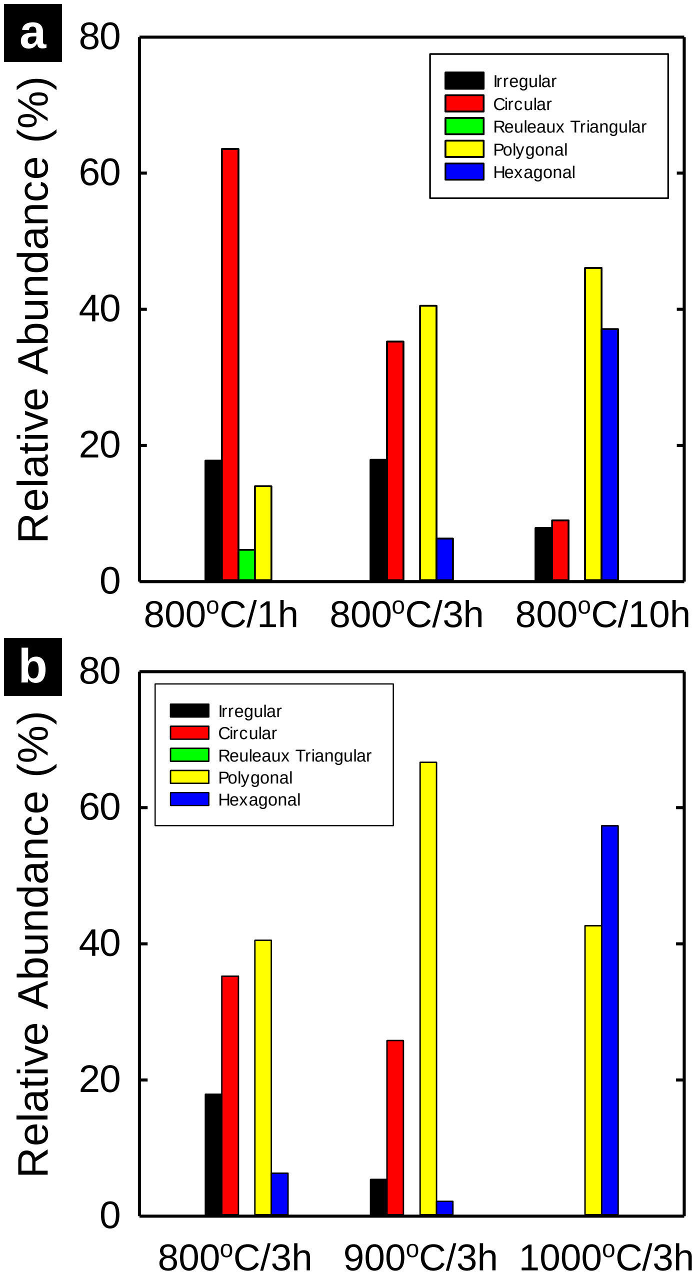


**Figure S5.** Statistical charts showing the evolution of various geometrical shapes of pits and holes for thermally treated Ag-BN: (a) comparison heating at 800 °C with duration of 1, 3, and 10 h, respectively; (b) comparison of 3 h heating at 800, 900, and 1000 °C, respectively. Samples from 900 and 1000 °C treatment were after acid purification.

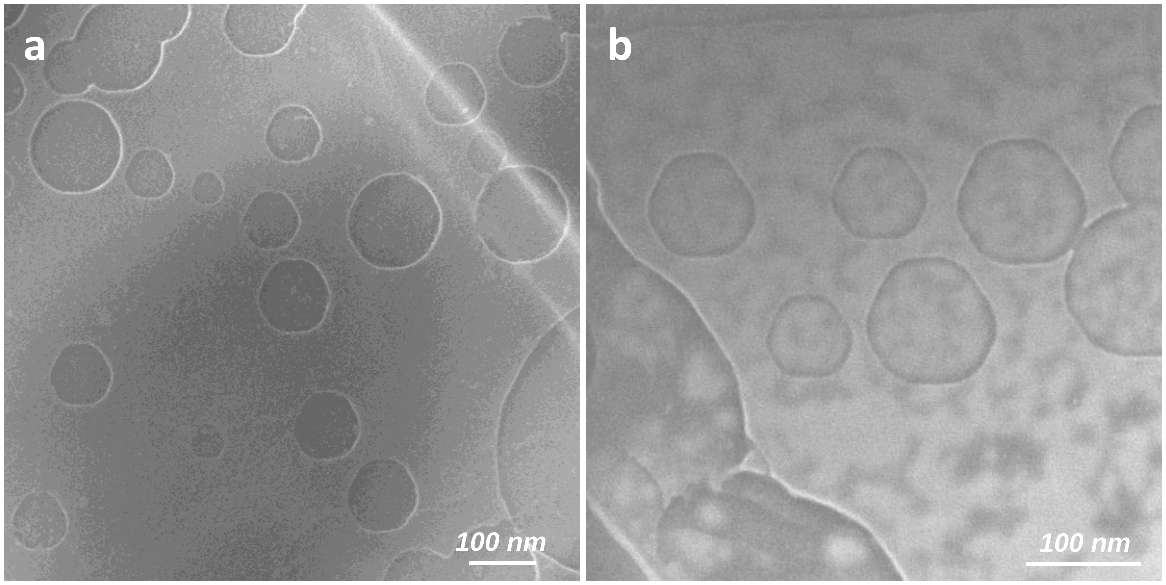


**Figure S6.** Typical SEM images of basal plane surface of acid-purified Ag-BN samples from etching at 800 °C for 3 h. Shown in (**a**) and (**b**) are multiple near-circular and near-hexagonal pits, respectively.

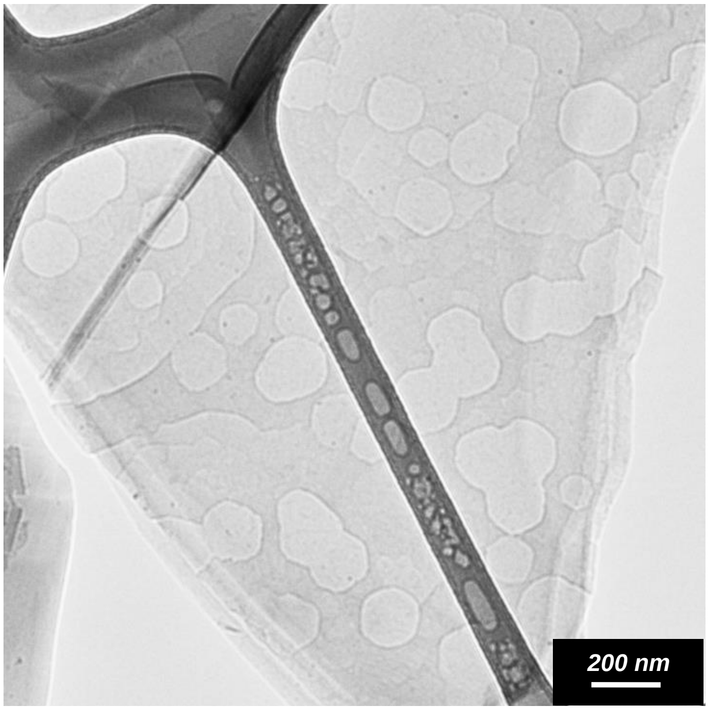


**Figure S7.** TEM image of a holey BNNS from the acid-purified Ag-BN sample (etching at 1000 °C for 3 h).

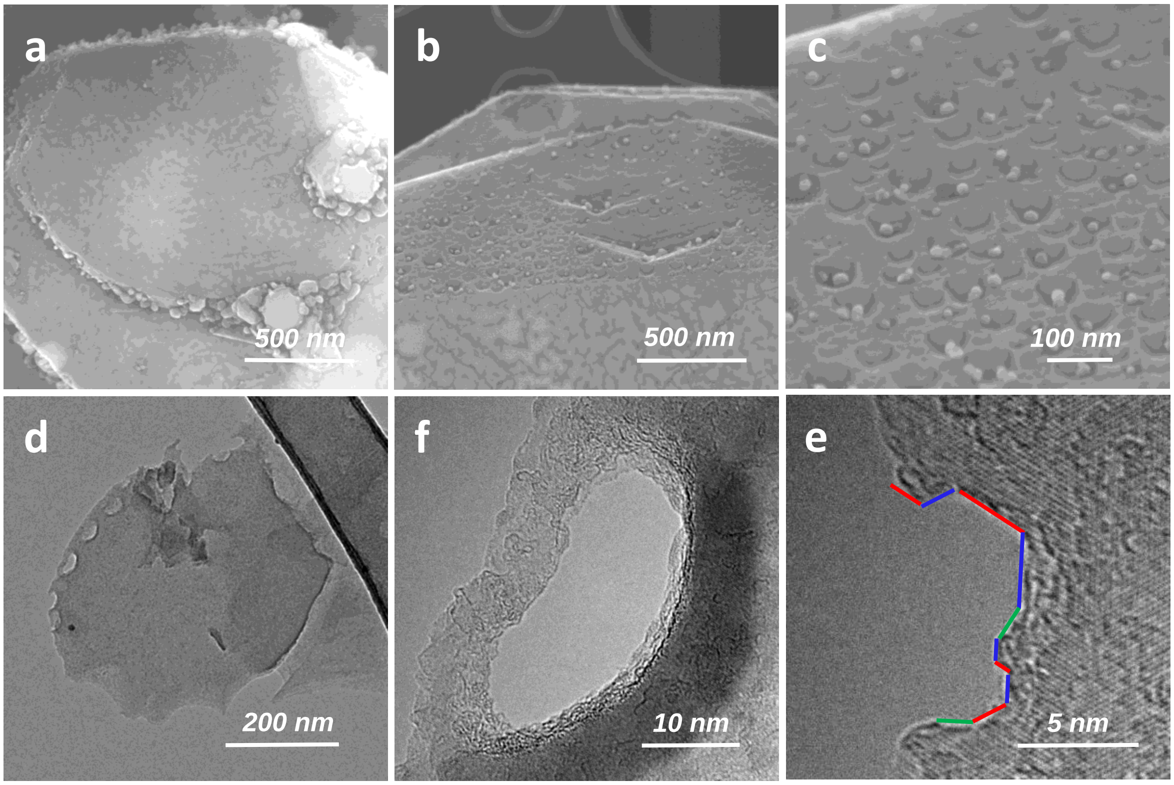


**Figure S8**. Perimeter-etched h-BN by heating the Ag-BN sample to 700 °C for 3 h: (**a-c**) typical SEM images of the as-obtained sample; (**d-f**) Typical TEM images of BNNS after removal of Ag using acid followed by solvent exfoliation using DMF. (**f**) is a partial circular shape; (**e**) is a polygonal shape with dominating *Z* edges. $Z_B$-, $Z_N$- and *A*-oriented edges are marked with red, blue, and green lines, respectively. Note the instrument resolution was insufficient to differentiate $Z_B$ vs. $Z_N$, thus the assignments of $Z_B$- and $Z_N$-oriented edges are interchangeable with $Z_N$- and $Z_B$-oriented edges, respectively.

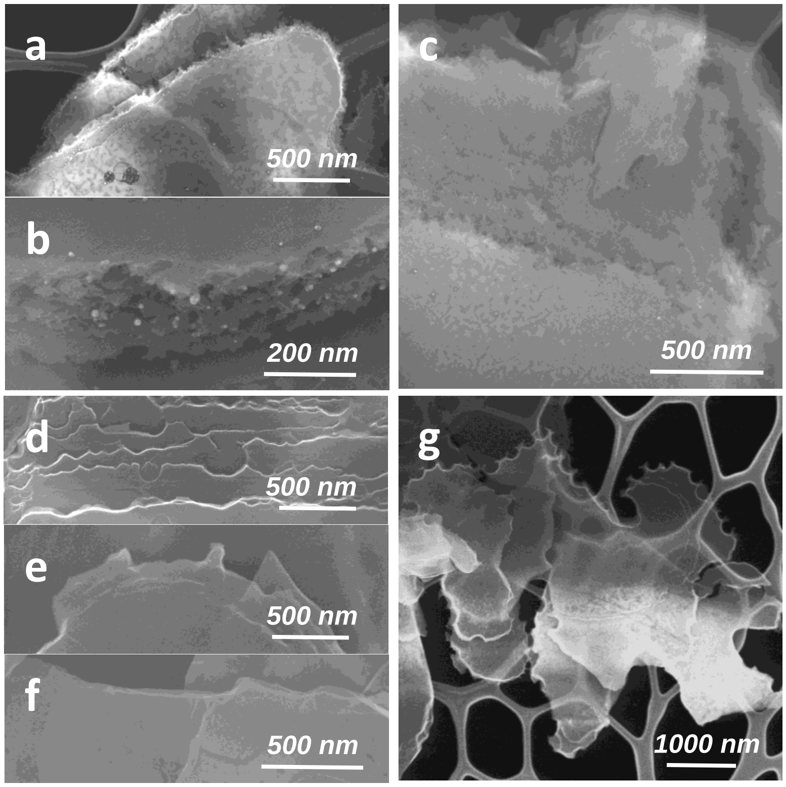


**Figure S9**. SEM images showing the perimeter etching of h-BN by heating the Ag-BN sample. (**a**) 800, (**b**) 900, and (**c**) 1000 °C, each with a duration of 3 h. (**d**) – (**f**) are the samples in the same order but after acid purification to remove Ag catalysts and the oxidative boron species. (**g**) is a low-magnification image of purified, etched h-BN from 800 °C/3 h etching.

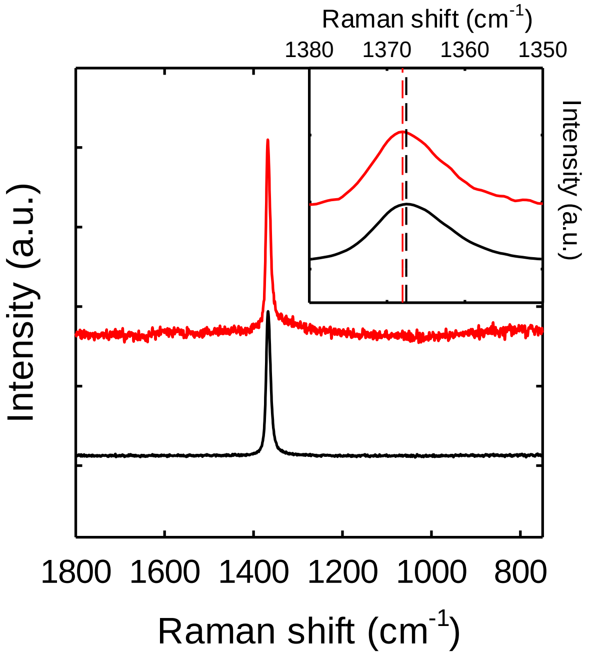


**Figure S10.** Raman spectra of BNNSs obtained from Ag-BN etched at 1000 °C followed by acid purification and DMF exfoliation (red) in comparison to DMF-exfoliated pristine BNNS (black). Inset shows the enlargement of the $E_{2g}$ peaks for the same spectra.

**Table S1.** Elemental composition (atom% from XPS) of as-prepared and etched Ag-BN samples.

| % | As-prepared | 700 °C | 800 °C | 900 °C | 1000 °C |
|---|---|---|---|---|---|
| **B** | 42.6 | 44.8 | 45.7 | 43.7 | 38.9 |
| **N** | 43.3 | 44.9 | 41.4 | 36.6 | 20.6 |
| **O** | 5.6 | 7.8 | 10.3 | 17.1 | 40.0 |
| **Ag** | 8.5 | 2.5 | 2.6 | 2.6 | 0.5 |
